# Cytochrome P450/ABC transporter inhibition simultaneously enhances ivermectin pharmacokinetics in the mammal host and pharmacodynamics in *Anopheles gambiae*

**DOI:** 10.1038/s41598-017-08906-x

**Published:** 2017-08-17

**Authors:** Carlos J. Chaccour, Felix Hammann, Marta Alustiza, Sandra Castejon, Brian B. Tarimo, Gloria Abizanda, Ángel Irigoyen Barrio, Helena Martí Soler, Rafael Moncada, José Ignacio Bilbao, Azucena Aldaz, Marta Maia, José Luis Del Pozo

**Affiliations:** 1 Instituto de Salud Tropical, Universidad de Navarra, Pamplona, 31008 Spain; 2 ISGlobal, Barcelona Ctr. Int. Health Res. (CRESIB), Hospital Clínic - Universitat de Barcelona, Barcelona, 08036 Spain; 3 Centro de Investigação em Saúde de Manhiça, Maputo, 1929 Mozambique; 4 Division of Clinical Pharmacology and Toxicology, University Hospital Basel and University of Basel, Basel, 4056 Switzerland; 5 Faculty of Medicine, Universidad de Navarra, Pamplona, 31008 Spain; 6 Department of Microbiology, Clínica Universidad de Navarra, Pamplona, 31008 Spain; 7 Ifakara Health Institute, Bagamoyo, Pwani P.O. Box 74, United Republic of Tanzania; 8 Centro de Investigación Médica Aplicada, Pamplona, 31008 Spain; 9 Drug Development Unit Universidad de Navarra (DDUNAV), Pamplona, 31008 Spain; 10 Department of Anaesthesia, Clínica Universidad de Navarra, Pamplona, 31008 Spain; 11 Department of Radiology, Clínica Universidad de Navarra, Pamplona, 31008 Spain; 12 Department of Pharmacy, Clínica Universidad de Navarra, Pamplona, 31008 Spain; 13 Swiss Tropical and Public Health Institute, Basel, 4051 Switzerland; 14 University of Basel, Basel, 4003 Switzerland; 15 Infectious Diseases Division, Clínica Universidad de Navarra, Pamplona, 31008 Spain; 16 KEMRI-Wellcome Trust Research Programme, Kilifi, Kenya

## Abstract

Mass administration of endectocides, drugs that kill blood-feeding arthropods, has been proposed as a complementary strategy to reduce malaria transmission. Ivermectin is one of the leading candidates given its excellent safety profile. Here we provide proof that the effect of ivermectin can be boosted at two different levels by drugs inhibiting the cytochrome or ABC transporter in the mammal host and the target mosquitoes. Using a mini-pig model, we show that drug-mediated cytochrome P450/ABC transporter inhibition results in a 3-fold increase in the time ivermectin remains above mosquito-killing concentrations. In contrast, P450/ABC transporter induction with rifampicin markedly impaired ivermectin absorption. The same ketoconazole-mediated cytochrome/ABC transporter inhibition also occurs outside the mammal host and enhances the mortality of *Anopheles gambiae*. This was proven by using the samples from the mini-pig experiments to conduct an *ex-vivo* synergistic bioassay by membrane-feeding *Anopheles* mosquitoes. Inhibiting the same cytochrome/xenobiotic pump complex in two different organisms to simultaneously boost the pharmacokinetic and pharmacodynamic activity of a drug is a novel concept that could be applied to other systems. Although the lack of a dose-response effect in the synergistic bioassay warrants further exploration, our study may have broad implications for the control of parasitic and vector-borne diseases.

## Introduction

In spite of remarkable advances since the turn of the century, malaria continues to be a major public health problem in most tropical countries^1^. Most of these recent advances can be attributed to the scale-up of vector control interventions such as long-lasting insecticidal nets (LLINs) and indoor residual spraying (IRS)^2^. Malaria is, however, a moving target and mosquito vectors do not cease to evolve and adapt in response to the pressure exerted by our control measures. The spread and intensity of insecticide resistance^3^ and behavioural adaptations that allow avoidance of insecticides and other home-centred control measures^4, 5^ are two of the major challenges faced by the malaria community today.

In this context, the mass use of drugs that can kill mosquitoes feeding on treated subjects has potential to become a new paradigm for vector control. These drugs, known as endectocides, could allow targeting of mosquitoes that avoid or are resistant to currently used insecticides and thus, could be a complementary intervention for malaria elimination^6, 7^. When modelling this potential intervention, the duration of the mosquito-killing effect is the parameter with the greatest impact on malaria transmission. The longer the drug is present in the blood, the larger the magnitude of effect will be^8, 9^.

Ivermectin is one of the most broadly studied endectocides. It effectively kills malaria vectors in the insectary^10^ and in the field^11, 12^. It has also been distributed to more than 2.5 billion people in the last 30 years for the control of onchocerciasis and other neglected tropical diseases (NTDs)^13^. For that use, it has an excellent safety profile^14^. Ivermectin, however, has a relatively short half-life of 18 hours^15^, which would limit the duration of the mosquito-killing effect. Concentrations that kill 50% of *Anopheles gambiae* in 10 days can only be sustained for around 72 hours after the single dose of 200 mcg/kg commonly used for NTDs; for other less susceptible species like *Anopheles aquasalis* ^16^, this mosquito-killing window can be shorter.

Several strategies have been proposed to overcome the relative short half-life of ivermectin and increase its potential impact on malaria transmission. These include using higher doses than approved for NTDs^17^, using repeated doses at regular intervals^18^ or developing slow-release formulations^8, 19^. The comparative advantages and disadvantages of each strategy has been described elsewhere^20^.

One additional strategy is to use a second drug to intentionally slow down ivermectin´s metabolism and elimination and boost plasma levels, sustaining them for longer periods of time. This is known as pharmacoenhancement and is commonly used in HIV treatment with protease inhibitors^21^.

Protease inhibitors, like many drugs including ivermectin^22^ are metabolised by the cytochrome P450 (CYP) 3A enzymes. The pharmacokinetic (PK) profile of the target drug is enhanced by adding either Ritonavir, a broad CYP inhibitor at doses below antiretroviral efficacy, or by using Cobicistat, a more specific CYP 3A4 inhibitor recently licensed for this purpose^21^. This approach reduces pill burden, improves adherence to treatment and spares active pharmaceutical ingredient of the boosted protease inhibitor^21^.

Modulation of the ABC transporter P-glycoprotein (P-gp) can also be used to improve the pharmacokinetic profile of certain medicines. The P-glycoprotein is an active efflux transporter; its action is normally protective by pumping out xenobiotics^23^. In humans, it is present primarily in endothelial cells with transport or barrier roles, such as intestinal mucosa or the capillaries in the blood-brain barrier^24^. In recent years, attempts have been made at reducing P-gp activity with the goal of increasing bioavailability and therapeutic benefit of certain drugs in humans, e.g. by better access to central nervous system targets or overcoming acquired P-gp-mediated resistance to chemotherapeutics^25–28^. Ivermectin is a substrate and an inhibitor of P-gp^29^.

One important challenge for using pharmacoenhancement strategies with ivermectin is the role of P-gp at the blood-brain barrier. Mice and dogs with a dysfunctional P-gp show increased susceptibility to ivermectin due to abnormal accumulation of the drug in the brain^30, 31^. There is a theoretical concern for this happening in humans, although supported by very little data^32, 33^. This potential for added toxicity has been evaluated in HIV and cancer therapy with encouraging safety results^34^.

We conducted a drug-drug-interaction study of ivermectin in a mini-pig model using ketoconazole, a broad CYP3A4 inhibitor. This drug was used for a first proof-of-concept step, given its capacity to inhibit both the CYP and the P-gp. Although it had been described in animal models that ketoconazole enhances systemic exposure to ivermectin^35, 36^, it is not completely clear whether this is done by reducing metabolism (directly related to CYP) or by reducing excretion (more related to P-gp inhibition).

Additionally, in the mosquito, metabolic resistance mechanisms drive an important proportion of insecticide resistance in Africa^37, 38^. There is no available data on the role of mosquito P450 in ivermectin detoxification; however, permethrin-resistant *Aedes aegypti* adults have a significantly increased ivermectin 5-day LC_50_ when compared with permethrin-sensitive counterparts^39^. Both compounds have different targets; this suggests a role of metabolic pathways involving P450s or xenobiotic pumps such as the P-gp.

If ivermectin is scaled up for vector control, this will exert selective pressure on mosquitoes, a process that can eventually lead to ivermectin resistance. In the face of the challenge posed by resistance to public health insecticides, a thorough understanding of the mosquito metabolic pathways and potential defence mechanisms from ivermectin can be pivotal if this novel strategy were to be used in the field. To date, ivermectin drug-class resistance in arthropods has been associated with a wide range of mechanisms: reduced cuticular penetration^40^, mutation of the glutamate-gated chlorine channel^41^ and metabolic resistance due to overexpression of xenobiotic pumps from the ABC family, like the P-gp^42–44^ and cytochrome P450 isoenzymes^44, 45^.

Since ketoconazole is a broad inhibitor of CYP and P-gp, our drug-drug interaction study provided a unique opportunity to test the concept of whether vector mortality is also enhanced by inhibition of both mechanisms in the mosquito as well. Using the original blood samples, we conducted a synergistic bioassay^46^ to evaluate the potential involvement of these mechanisms in the metabolism/detoxification of ivermectin in *Anopheles gambiae* and whether this information could be harnessed to enhance the effect of the drug on the vector.

Our main aims were: (a) to assess the PK modifications induced by a dual CYP/P-gp inhibitor, (b) to assess the safety of ivermectin in the presence of P-gp inhibition in a mini-pig model, (c) to determine the effect of ketoconazole alone on mosquito mortality and (d) to determine whether ketoconazole by means of CYP/P-gp inhibition increases the ivermectin-driven mosquito mortality.

## Results

### Design of drug-drug interaction study

**Figure 1:**
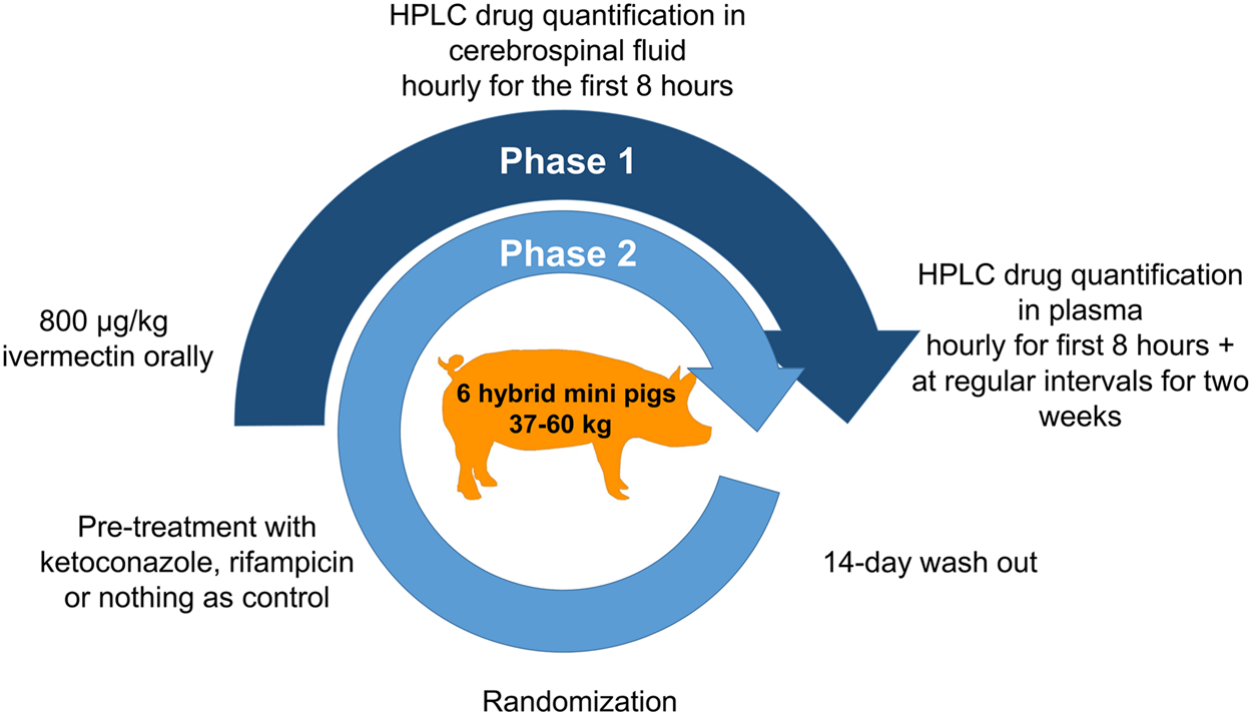
Illustration of the randomized-crossover design used in the study. In phase I all six pigs received a single 800 mcg/kg dose of oral ivermectin. After a washout period of 14 days, the animals were randomly assigned to pre-treatment with ketoconazole, rifampicin or nothing in a 1:1:1 ratio and ivermectin dosage and sampling was repeated. The time between ivermectin doses was 30 days. Figure by Juliane Chaccour.

The experiments were conducted following a randomised crossover design (Fig. 1). In phase I all mini-pigs received a single 800 mcg/kg dose of oral ivermectin and sampling was done as described below. After a washout period of 14 days, the animals were randomly assigned to pre-treatment with either ketoconazole (200 mg daily, orally, for 14 days), rifampicin (10 mg/kg daily, orally, for 14 days) or nothing in a 1:1:1 ratio and ivermectin dosage and sampling was repeated. The total wash-out period between ivermectin doses was 30 days. Ketoconazole is a dual inhibitor of the CYP and the P-glycoprotein (P-gp), which plays a key role as xenobiotic pump in the blood-brain barrier and other epithelial barriers^23, 24^. Inhibiting the P-gp could theoretically lead to toxicity due to abnormal accumulation of the drug in the brain^30, 31^. The dose of 200 mg/day was calculated to maximise the time ketoconazole remained above the 50% inhibitory concentration (IC_50_) for the CYP 3A4 (estimated in 0.022–0.025 µM [1.6–13.2 ng/ml]^47^) but remaining below the much higher IC_50_ for the P-gp (estimated in 5.6 µM [2975 ng/ml]^48^). Irrespectively of this, drug quantification in cerebrospinal fluid (CSF) was added as an additional safety point. Rifampicin a CYP/P-gp inducer^49^ was included as we expected to see reduced bioavailability and penetration into CSF.

The main outcome measure was time above the ivermectin concentration that kills 50% *Anopheles gambiae* in 10 days [10-day lethal concentration 50 (LC_50_)], which has been estimated at 6 ng/ml^50^. Secondary outcome measures were the maximum concentration reached (Cmax), the total area under the curve (AUC) and the area under the curve above the LC_50_ (AUC > LC_50_).

### Design and sample size of mosquito bioassay

Mosquitoes were membrane fed with reconstituted blood as described by Bousema *et al*.^51^. Feedings were done with samples drawn directly from the pigs at different intervals and pair-matched according to ivermectin concentration, thus the only different between the samples in a pair was the presence or absence of ketoconazole.

Since mosquitoes grouped in cups for feeding, we used a cluster design in which the unit of intervention was mosquito cups. Our primary outcome was mean 10-day mosquito mortality after membrane feeding. Sample size calculations were conducted according to the method described by Gangnon and Kosorok^52^. The minimum expected 10-day mortality in mosquitoes imbibing blood containing 9.9 ng/ml of ivermectin is 66%; this is based on the predictive function described by Ouedraogo *et al*.^50^. Based on previous observations and with all mosquitoes having the same colony origin and age range, we chose a conservative intracluster correlation of 0.15. The frailty variance of clusters was resembled by a gamma of 1. All experiments were performed in triplicate. With these parameters, at least 4 clusters of 44 mosquitoes were needed per arm to achieve 80% power at 5% significance level accounting for 10% non-feeders.

After determining of the ivermectin levels in all samples in the drug-drug interaction study, we selected 4 paired samples with matching ivermectin concentrations (+/−1 ng/ml) from naïve and ketoconazole pre-treated groups. Baseline serum samples collected before ivermectin treatment in ketoconazole and naïve pigs were used as controls. These samples were shipped frozen to the insectary facilities of the Ifakara Health Institute in Bagamoyo, Tanzania for membrane feeding assays. It was hypothesised that induction of the mosquito CYP/P-gp by rifampicin could improve survival in presence of ivermectin, but this could not be tested due to a reduced absorption of ivermectin in the mini-pigs pre-treated with rifampicin (see below).

### Pharmacoenhancement

Ivermectin in both phases and pre-treatments in phase II were administered uneventfully to all subjects. Blood samples were obtained according to the planned schedule. Lumbar punctures were performed with mixed success. It was possible to insert a tunnelled intrathecal catheter and reservoir in 5 out of 6 pigs. Two reservoirs were removed within 48 hours due to motor impairment with full recovery after removal. One pig (#72) was euthanised 72 hours post procedure due to non-reversible motor impairment and was replaced by a seventh animal (#75). At least one CSF sampling point was obtained from 5 out of 6 pigs in phase I. The success rate was considerably higher during phase II with CSF samples obtained from 6 out of 6 pigs.

### Ketoconazole enhances ivermectin’s PK

The main plasma PK parameters in all groups are presented in Table 1. The plasma PK parameters of ivermectin-naïve pigs were compatible with previously published data^53, 54^.

**Table 1:** Relevant PK parameters by treatment group.

Treatment	Subject	Cmax [ng/mL]	AUC inf [h·ng/mL]	Time > LD50 [h]	AUC > LD50 [h·g/mL]
Ivermectin 1	69	14.3	1916.2	46.7	509.4
	70	24.1	694.9	27.2	198.9
	71	3.0	16.9	0.0	0.0
	72	1.6	—	0.0	—
	73	27.6	244.3	9.3	47.3
	74	2.9	99.4	0.0	0.0
	**Median (Range)**	**8.7 (26.0)**	**244 (1899)**	**4.6 (46.7)**	**23.7 (509.4)**
Ivermectin 2	73.2	15.2	1380.0	54.5	226.3
	75	20.9	1030.5	45.4	368.9
	**Median (Range)**	**18 (5.6)**	**1205.3 (349)**	**49.9 (9.1)**	**297.6 (142.6)**
Ivermectin(combined)	69	14.3	1916.2	46.7	509.4
	70	24.1	694.9	27.2	198.9
	71	3.0	16.9	0.0	0.0
	73.2	15.2	1380.0	54.5	226.3
	74	2.9	99.4	0.0	0.0
	75	20.9	1030.5	45.4	368.9
	**Median (Range)**	14.8* (21)	**862.7 (1899)**	36.3* (54)	212.6* (509)
Ivermectin + ketoconazole	69	27.7	2320.7	117.1	703.9
	72	28.4	1863.3	104.6	718.5
	**Median (Range)**	28.0* (0.7)	**2092 (457.4)**	110.8* (12.4)	711.2* (14.6)
Ivermectin + rifampin	71	0.3	—	0.0	0.0
	74	6.8	—	4.7	—
	**Median (Range)**	**3.6 (6.5)**	—	**2.4 (4.7)**	—

Footnote: Cmax: peak plasma concentration, AUC inf: area under the PK curve to infinity, LD50: the 10-day lethal concentration 50 of ivermectin for *Anopheles gambiae* (6 ng/ml). Pig 72 died and was replaced by pig 75. Pig 73 received ivermectin in phase 1 and was randomised to ivermectin alone in phase 2, this is denoted as 73/73.2, these observations were not combined in order to sustain sample independence. *Denotes a statistically significant difference.

Firstly, we compared the PK parameters between ivermectin-naïve pigs and those who received it in phase II without pre-treatment by means of a Mann-Whitney test and found no difference in time above LC_50_, Cmax, AUC or AUC > LC_50_ (P values of 0.12, 0.61, 0.16 and 0.32 respectively). Given these results, the small sample size and relative large variations between subjects (as expected in a mini-pig model), these two groups (excluding one animal for which lamda could not be calculated) were combined for comparisons between ivermectin alone and ketoconazole/rifampicin pre-treated pigs. In order to respect sample independence, only the values of the second treatment of mini-pig 73 were used, this penalized the main outcome by increasing the time above LC_50_ of the combined ivermectin.

**Figure 2:**
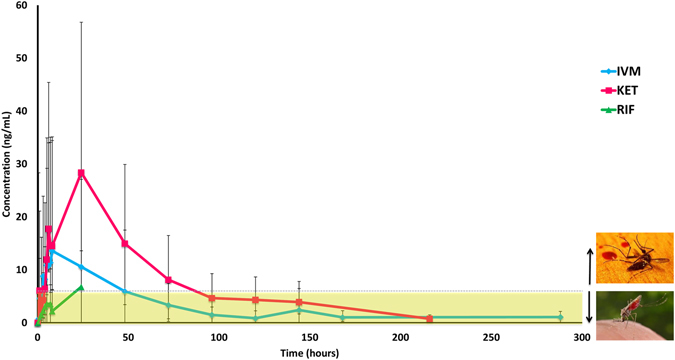
Comparison of median (±range) plasma concentrations across different treatment groups. The dashed line indicates the target 10-day lethal concentration (LC_50_) for *Anopheles gambiae*. Alive *Anopheles* image in public domain from CDC Public Health Image Library, photo credit: James Gathany. Dead mosquito image CC-by-sa PlaneMad/Wikimedia available at https://commons.wikimedia.org/wiki/File:Dead_mosquito.jpg. CDC: Centers for Disease Control and Prevention, IVM: ivermectin, KET: ketoconazole, RIF: rifampicin.

The PK curves of all three groups (ivermectin alone, ketoconazole + ivermectin and rifampicin + ivermectin) are shown in Fig. 2.

Pre-treatment with ketoconazole significantly increased the time above LC_50_ 3-fold from a median of 36 to 111 hours (P = 0.033). The Cmax was also increased 2-fold (median 14 vs 28 ng/ml, P = 0.033), along with a more than 3-fold increase in the AUC > LC_50_ (median 212 vs 711, P = 0.033). The total AUC was also increased more than 2-fold (862 vs 2092 h*ng/ml) but this difference was not statistically significant (P = 0.067).

Pre-treatment with rifampicin seriously hampered ivermectin absorption making the AUC and AUC > LC_50_ calculations uninterpretable. Only one pig reached ivermectin plasma concentration above 6 ng/ml, which was maintained for less than 5 hours in total (Fig. 2).

### Pre-treatment with ketoconazole did not increase ivermectin in the CSF

No clinical adverse events were observed. Although detectable in some, ivermectin was below quantification level (0.5 ng/ml) in all CSF samples obtained (Table 2). Of 24 CSF samples available from phase I, ivermectin was detectable in 4. Of 37 CSF samples available from phase II, ivermectin was detectable in 4 samples (2 in the ketoconazole group and 2 from rifampicin group).

**Table 2:** Number of CSF samples with detectable and quantifiable ivermectin divided by study phase and treatment group.

Concentration	**Phase I**	**Phase II**		
	**Ivm**	**Ivm**	**Ket**	**Rif**
< 0.1 ng/ml	20	10	10	13
> 0.1 < 0.5 ng/ml	4	0	2	2
**Total**	**24**	**10**	**12**	**15**

Footnote: Ivm: ivermectin only group, Ket: ketoconazole + ivermectin group, Rif: rifampicin + ivermectin group.

### Mosquito bioassay

Out of a total of 169 samples available we selected four pairs with matching ivermectin concentrations (+/−1 ng/ml) that only differed in the presence of ketoconazole, plus two controls; one from a ketoconazole-only and one from a fully untreated pig (Table 3). In the rifampicin group, there was only one sample with matching ivermectin concentration in naïve pigs.

**Table 3:** Mean survival and time to median mortality of mosquitoes feeding on blood samples with matching concentrations (+/−1 ng/ml) of ivermectin with (K) or without ketoconazole at CYP inhibition concentrations.

Pair	Drug concentrations	Mean survival (days)	Time to median mortality (days)
**1**	**IVM 9.98**	6.73 (6.29–7.17)	7
	**KET 8.96**	5.39 (4.90–5.89)	5
**2**	**IVM 16.5**	5.11 (4.63–5.86)	5
	**KET 17.23**	3.79 (3.49–4.09)	4
**3**	**IVM 20.46**	7.43 (7.11–7.75)	7
	**KET 20.39**	5.10 (4.51–5.69)	4
**4**	**IVM 27.58**	5.28 (4.54–6.02)	5
	**KET 27.68**	4.23 (3.80–4.65)	4

Footnote: IVM: ivermectin alone group, KET: ketoconazole + ivermectin group.

### Ketoconazole is not lethal to mosquitoes at the dose used

Firstly we assessed whether the ketoconazole concentration present in the samples could have a lethal effect *per se*. For this we compared the 10-day mortality of mosquitoes fed on samples containing only ketoconazole (baseline samples from ketoconazole pre-treated pigs) with the mortality of mosquitoes feeding on fully drug-free samples (baseline of control pigs). Given that all pigs received ivermectin in the first phase, this experiment served as well to assess whether there could be active metabolites increasing mortality in spite of a washing period of 30 days.

**Figure 3:**
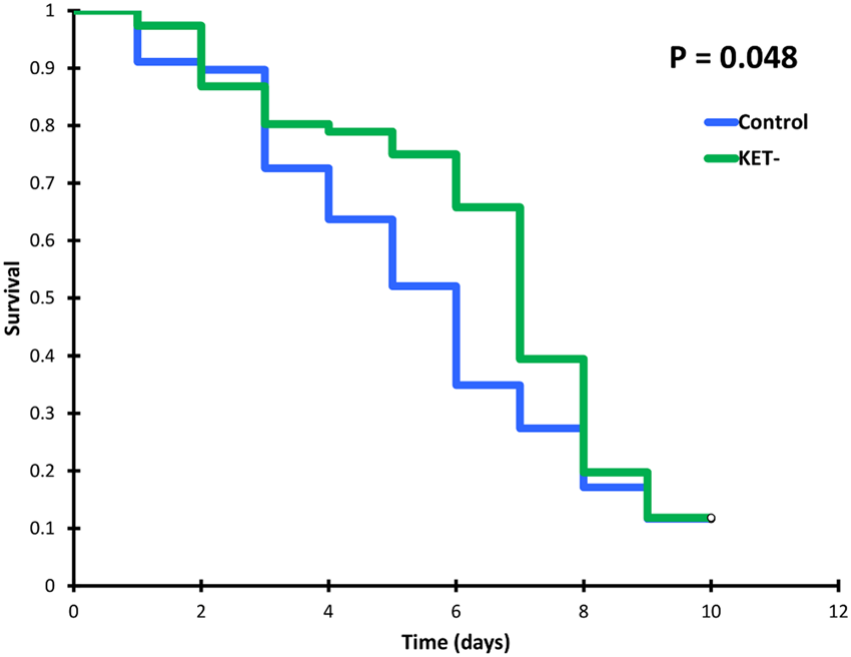
Survival curves of mosquitoes feeding on blood from one fully untreated pig (control) vs mosquitoes feeding on a ketoconazole-only treated pig (Ket-). Triplicate experiments, n = 222.

Ketoconazole alone did not increase the mortality of *Anopheles gambiae*, in fact it provided a slight, protective effect (Long-rank P = 0.048, n = 222), as previously seen with other antimicrobials^55^ (Fig. 3).

### Ketoconazole synergises the mosquito mortality caused by ivermectin

**Figure 4:**
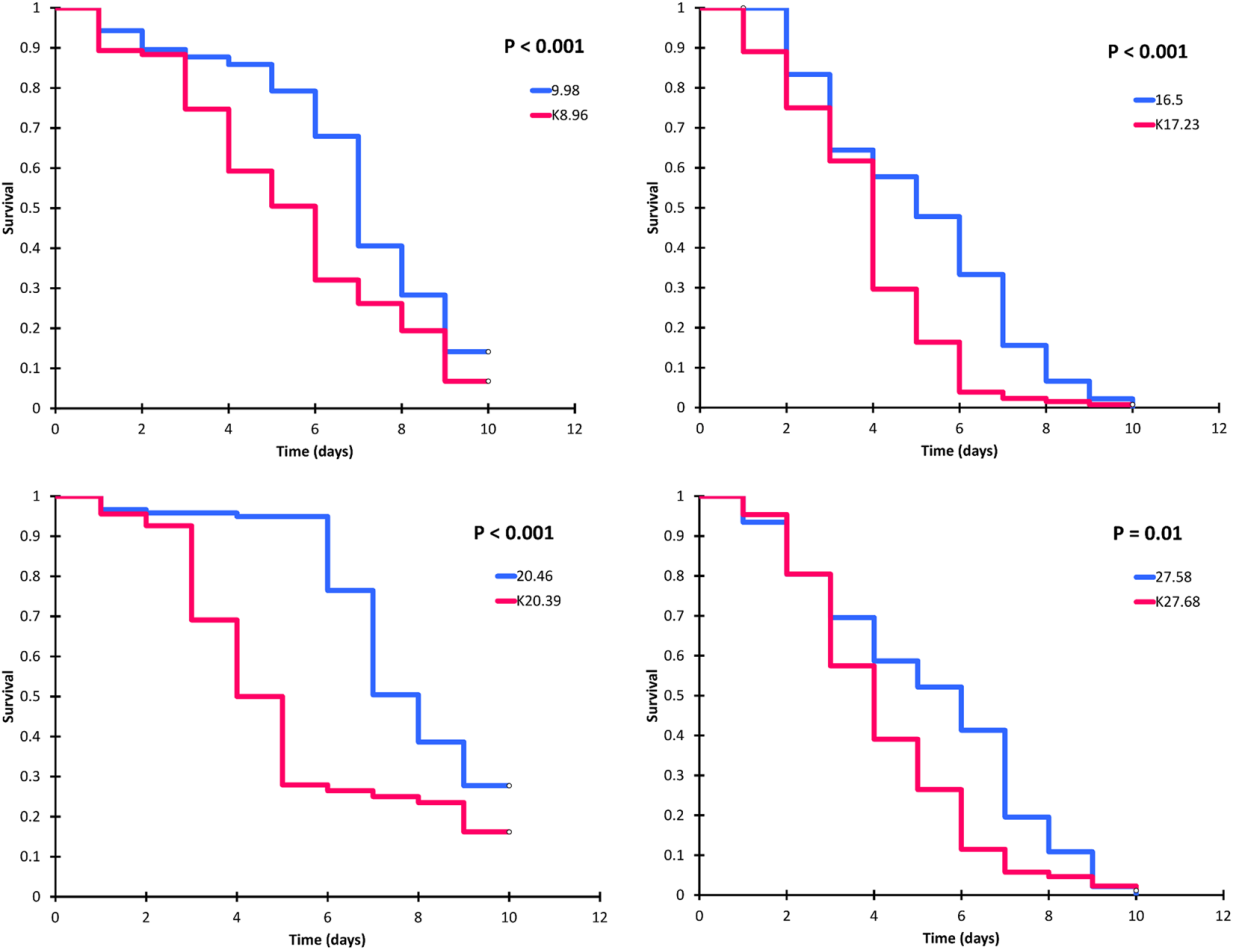
Survival curves of mosquitoes feeding on blood with matching ivermectin concentrations (+/−1 ng/ml) with (blue) or without (pink) the ketoconazole at CYP inhibition concentrations. Triplicate experiments, n = 133–220.

Ketoconazole significantly increased the mortality of *Anopheles gambiae* fed in all samples containing ivermectin irrespectively of the ivermectin concentration (Fig. 4). The effect was predominantly observed at the expense of early mortality as seen by a reduction in the mean survival and the time to median mortality in all ketoconazole groups (Table 3).

The mortality of mosquitoes fed on the only paired sample with rifampicin-ivermectin did not differ from that of ivermectin or rifampicin alone groups (data not shown).

## Discussion

Our study shows that inhibition of the CYP 3A4 could safely increase ivermectin’s time above target insecticidal concentration in the mammal host, increasing mosquito exposure. This finding could be harnessed to simplify the dosing regime and spare active pharmaceutical ingredient. Additionally, once outside the mammal host, pharmacological CYP inhibition increases the mortality of exposed *Anopheles gambiae* mosquitoes, which could further improve efficacy. A natural next step would be to assess the potential implications of these findings in the field by means of modelling studies.

CYP inhibitors that also target the P-gp could theoretically increase penetration of the drug into the CNS and facilitate interaction with the GABA receptors, to which ivermectin has weak affinity. Thus, our main concern was that ketoconazole mediated CYP inhibition might reduce ivermectin’s excellent safety profile. Our results suggest that the large therapeutic window of ivermectin and the difference in IC_50_ for CYP and P-gp could safely allow CYP inhibition without a measurable increase of the drug in the CSF or clinically observed adverse events. Currently there are some “selective” CYP3A4 or P-gp inhibitors on the market but most drugs that affect one system typically have at least a partial effect on the other. Emerging more selective molecules^56^ or even combination strategies^34^ could overcome this issue.

Ivermectin resistance resulting from scale up of its veterinary use is well known. It was observed in nematodes as early as 1985^57^, four years after licensure of the drug. It was soon proven that resistance could be selected in the lab after only 8 generations of *Haemonchus contortus* exposed to sub-optimal dosing^58^. In arthropods highly ivermectin-resistant *Musca domestica* could be selected in the lab after only seven generations in the early 1990s^59^. Field reports in economically or public health relevant arthropods followed shortly^60, 61^. The relative delay in global resistance reports was the result of ivermectin’s broad spectrum. Most intestinal parasites in this spectrum could be removed with doses as low as 20 mcg/kg, but the 200 mcg/kg dose was selected based on the less susceptible organisms, the dose-defining species. The result is that any resistance arising in the more susceptible parasites could not be detected until their susceptibility was 10-fold higher^62^. This phenomenon has been called the window of escalation^62^ and could be an important concept it if ivermectin is scaled up for vector control given that malaria vectors also differ in their ivermectin susceptibility^16, 63, 64^. While *Anopheles gambiae* ^50^ and *Anopheles minimus* seems to be remarkably susceptible based on their low LC_50_, others like *Anopheles aquasalis* or *Anopheles dirus* promise to become the dose-defining mosquito species^16, 63, 64^ for the novel vector control use of the drug. Taking advantage of a synergist could potentially delay the emergence of ivermectin resistance in malaria vectors and extend the spectrum of ivermectin towards less susceptible vectors.

The findings from our study suggest that although metabolic pathways play a role in the mosquito defence from ivermectin, the activity of the detoxification mechanisms or efflux pumps could be modulated to synergise ivermectin and boost its efficacy. These findings warrant surveillance of early signs of metabolic resistance if ivermectin is used to reduce malaria transmission. At the same time, these results offer a simple potential tool to address metabolic resistance before it challenges efficacy.

Additionally, emerging data indicates that active ivermectin metabolites could cause mosquito mortality even when the mother drug is no longer detectable in plasma^65, 66^. Increasing the time above mosquitocidal levels of ivermectin at the expense of reducing active metabolites could leave overall mosquito mortality unchanged^20^. Previous animal studies suggest dual CYP3A4/P-gp inhibitors do not significantly modify the PK of one of the major ivermectin metabolites, 3 O-desmethyl ivermectin^35, 36^ potentially suggesting an increase in systemic exposure mostly due to reduced excretion rather than reduced metabolism. Nonetheless, the evaluation of ivermectin metabolites is a complex process^22, 67^ and their potential role in the insecticidal effect of the drug would require additional investments.

Ketoconazole was used purely to test the concept of dual CYP/P-gp inhibition enhancing the PK and the ivermectin-related mosquito mortality, but it is in no way a molecule suitable for combination in MDA campaigns. Some additional limitations of this study include: a small number of mini-pigs was used for the pharmacoenhancement experiment, which explains the relative large concentration ranges; this pharmacokinetic effect, however, has previously been seen in other animal models^35, 36^. We included rifampicin, a dual CYP3A4/P-gp inducer^49^ to validate the results, evaluate changes in the CSF concentration and better understand the potential routes of manipulation of detoxification enzymes/transporters in the mosquito. Yet, Rifampicin mediated CYP 3A4/P-gp induction led to a marked reduction in systemic ivermectin exposure, possibly due to reduced intestinal absorption and/or pre-systemic metabolism; Although there is no evidence that ivermectin is a substrate of Organic Anion-Transporting Polypeptides (OATPs) in hepatic tissue or the intestinal epithelium, decreased bioavailability when co-administered with orange juice suggests this could occur^68, 69^. If this was the case, inhibition of OATPs mediated uptake by rifampicin^70^ could have also contributed to the reduced ivermectin absorption seen in our rifampicin pre-treated mini-pigs. Rifampicin-ivermectin samples were insufficient for the mosquito experiment. Nonetheless, the role of MDR1 polymorphism on the pharmacokinetics and pharmacodynamics of ivermectin in humans requires further exploration^32, 33^, particularly in the African setting^71, 72^. Finally, we acknowledge the lack of a dose-response effect in the membrane feeding assays. These assays were performed with reconstituted blood which can lead to varying final drug concentrations given to mosquitoes caused by the manual mixture; this could explain the apparent absence of a dose-response effect in our experiments but does not affect our conclusion about a pharmacodynamic synergism. Future experiments assessing drug concentrations inside the mosquito could help clarify this potential source of bias.

When used as anthelmintics, dual pharmacokinetic and pharmacodynamic enhancement of macrocyclic lactones such as ivermectin can occur by modulation of the ABC transporter. This results in higher drug bioavailability in the host and higher drug penetration in the nematode^73, 74^, which is the main driver of efficacy. However, this dual *in vivo* enhancement by inhibiting the CYP has not been reported, even though it could have important implications for the treatment of certain helminths^75–78^. Particularly in the context of wider use of ivermectin mass-treatment as resistance in endo- and ectoparasites can be induced simultaneously^79^.

Provided an appropriate regimen of a selective molecule is defined and the appropriate cost-effectiveness evaluations are conducted, our findings could be applied to other systems and have important implications for the control of different vector-borne diseases.

## Materials and Methods

### Mini-pig procedures

The pre-medication drugs were administered by crushing the tablets and mixing them with canned meat products used for enrichment. For ivermectin administration and sampling during the first 8 hours, the animals were sedated with a single intramuscular dose of tiletamine-zolazepan (4 mg/kg) and maintained with inhalatory isoflurane (1.5–2%). In pigs, there is no evidence of a significant interaction (other than being a substrate) of these drug classes with the CYP3A4 or the P-gp^80^. Ivermectin was administered via nasogastric tube.

For blood sampling during the first eight hours, a femoral line was left in place and later removed during recovery from anaesthesia. Blood sampling thereafter was done by jugular puncture under restrain. Sampling points for blood were: pre-treatment, at 0.5, 1, 2, 3, 4, 5, 6, 7, 8, 24, 48/72 hours and at days 6/7, 9 and 14. For the mosquito bioassay, additional serum samples were obtained at every time point, and frozen at −20 °C.

**Figure 5:**
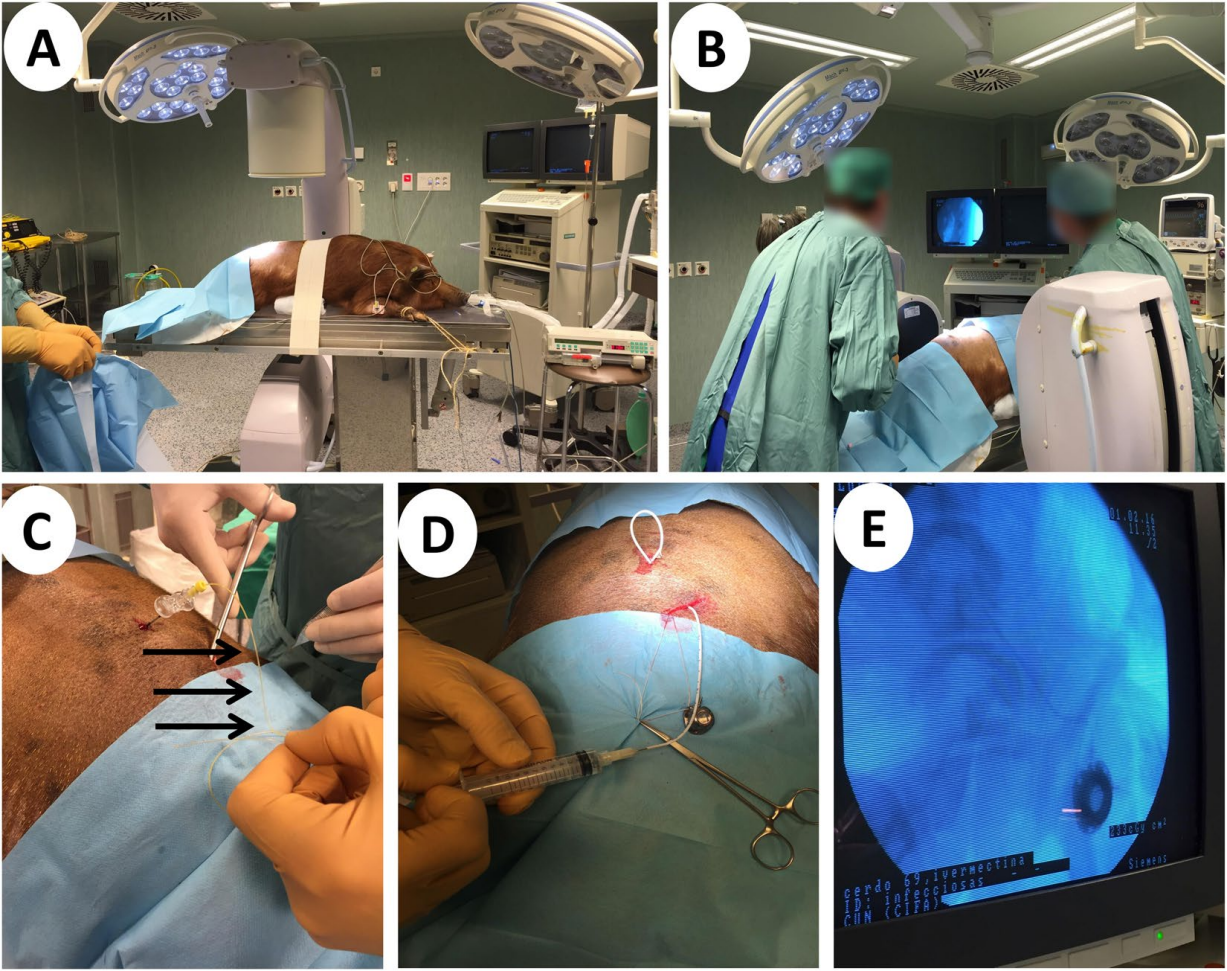
Cerebrospinal fluid sampling. (**A**) The pigs were sedated with tiletamine-zolazepan, intubated and maintained with inhalatory isoflurane. (**B**) Lateral lumbar puncture was performed in aseptic conditions by an anaesthetist and an interventional radiologist under fluoroscopic guidance. (**C**) After introduction of the intrathecal catheter (black arrows), it was tunnelled (**D**) and connected to a reservoir placed in subcutaneous tissue (**E**) to avoid a second round of laborious localization of the intrathecal space.

For obtaining CSF samples during the first 8 hours, an intrathecal catheter was placed by means of fluoroscopy-guided lateral lumbar puncture^81^ (Fig. 5). Given the challenges posed by the particular anatomy of the pig, efforts were done to tunnel the intrathecal catheter and connect it to a subcutaneous reservoir in order to avoid repeating the technically demanding lumbar puncture during phase II of the study (Fig. 5). Given the risk of damaging nerve roots when accessing the reduced thecal space, the pigs received at least 20 ml/kg of isotonic IV fluids during the first 8 hours and motor impairment lasting more than 72 hours was included as a humane endpoint criterion. Sampling points for CSF were: pre-treatment and at 0.5, 1, 2, 3, 4, 5, 6, 7 and 8 hours.

### Mosquito procedures

For all experiments, we used the fully susceptible *Anopheles gambiae* s.s. colony maintained at the insectary in Bagamoyo. Mosquitoes were reared at 28 °C, 80% humidity and with a photoperiod of 12:12. Larvae were kept in large plastic bowls covered with netting containing approximately 2 litres of water with larval densities not exceeding one 4^th^ instar larvae per ml. Larvae were fed Tetramine fish flakes *ad libitum*. Once they developed into pupae these were collected using disposable plastic Pasteur pipettes and transferred to small bowls containing approximately 200 ml of clean water. The pupae bowls were then placed inside fully screened cages (30 × 30 × 30 cm) where adult mosquitoes were allowed to emerge and given 10% glucose as a sugar source *ad libitum*.

Only mosquitoes of 2–5 days of age were used for the experiments. Before the experiments, mosquitoes were starved from sugar for 12–18 hours and from water for one hour. Hungry females were selected by applying a bottle with heated water (37–40 °C) to the cage. 40–50 hungry females were transferred to each paper cup using a mouth aspirator.

We performed membrane feeding assays with serum replacement as previously described^51^. For each experiment, the selected pig sample was thawed and 1 ml of whole blood was centrifuged at 2100 rpm for 10 minutes. The serum volume was noted and replaced with the same amount of the sample pig-serum being tested. After this, tubes were inverted 10 times. Each sample was tested in triplicate.

In order to reduce potential confounding arising from variations in bloodmeal size, partially fed mosquitoes were discarded and only fully engorged females followed up for the mortality assessment. After each feeding experiment, the cups were placed on ice for 1–2 minutes to select and transfer fully engorged females.

Cups with fully engorged mosquitoes were kept at 24–26 °C and 65–70% relative humidity inside climate controlled incubators. Mosquitoes were maintained with 10% glucose solution using impregnated cotton. Mortality was recorded daily for 9 days by a person blinded to the allocation of the cups. Dead mosquitoes were removed from the cups daily.

### Analytics and PK calculations

Blood samples were drawn in 3 ml EDTA tubes and centrifuged. Plasma was separated and frozen at −20 °C until analysis. CSF samples were frozen at −20 °C.

Ivermectin levels in plasma and CSF were determined using a validated adaptation of a previously described HPLC-FLD^82^. The detection limit in plasma and CSF was 0.1 ng/ml, the quantification limit was 0.5 ng/ml.

We used the lethal concentration 50 of *Anopheles gambiae* described by Ouedraogo^50^ (6 ng/ml) as target concentration and particularly aimed at increasing the time ivermectin was present in plasma above this level. Pharmacokinetic analyses were performed using Phoenix WinNonlin 6.4 (Certara, Princeton, NJ, USA). Pharmacokinetic parameters were derived from non-compartmental analyses.

### Animals

Six hybrid mini-pigs (2 male, 4 female) weighing between 46 and 70 kg were procured from our accredited breeding centre at the University of Navarra. They were housed individually throughout the study at the animal research facilities in the University of Navarra. From arrival onward, the animals were assessed daily for general wellbeing and after the intervention, also for specific ivermectin toxicity signs.

### Drugs and reagents

Ivermectin (Noromectin® 0.08% oral solution, Norbrook) and ketoconazole (Fungiconazol 200 mg dog tablets, Fatro) were procured from our veterinary supplier. Rifampicin (Rifaldin® 300 mg tablets, Sandoz) was procured through the hospital pharmacy service.

European pharmacopeia standard ivermectin for HPLC calibration was procured from Sigma Aldrich.

### Statistics

The comparison of PK parameters between ivermectin groups was done by means of a two-sided Mann-Whitney test. Comparison between combined ivermectin and ketoconazole plus ivermectin groups was done with a one sided Mann-Whitney test.

Survival Kaplan-Meier analysis was performed in Addinsoft’s XLSTAT ® Version 2016.04.32229 (New York, NY, USA). Comparisons of survival patterns were done with Log-rank test using a 5% significance level adjusted for cluster design.

### Ethics

All procedures were reviewed and approved by the animal experimentation ethics committee of the Universidad de Navarra (Registry number E27-15(135-12E4)). All procedures were performed in accordance with the relevant guidelines and regulations.

### Data availability

The datasets generated during the current study are available from the corresponding author on reasonable request.
